# Comparison of arthrodesis and arthroplasty of Chinese thumb carpometacarpal osteoarthritis

**DOI:** 10.1186/s13018-019-1469-2

**Published:** 2019-11-29

**Authors:** Jianfeng Li, Dacun Li, Guanglei Tian, Wentong Zhang

**Affiliations:** Department of Upper Limb Surgery, Beijing Shunyi District Hospital, No.3 guangming south street, shunyi district, Beijing, 101300 People’s Republic of China

**Keywords:** Thumb, Carpometacarpal joint, Arthrodesis, Arthroplasty

## Abstract

**Background:**

The thumb carpometacarpal (CMC) osteoarthritis is very common. Multiple methods are used to treat progressive thumb CMC osteoarthritis, among which trapeziometacarpal arthrodesis and trapezial excision with ligament reconstruction and tendon interposition (LRTI) are the most common. These two surgical treatment methods have received mixed reviews in previous studies in the west patients. This retrospective study studied the effects, advantages, and disadvantages of arthrodesis and arthroplasty for treating thumb carpometacarpal osteoarthritis in Chinese patients.

**Methods:**

Between February 2012 and September 2017, 39 Chinese patients with stage II or III thumb carpometacarpal osteoarthritis underwent surgery (trapeziometacarpal arthrodesis in 22, trapezial excision with ligament reconstruction and tendon interposition in 17). Postoperative objective and subjective evaluations were performed. The objective evaluation involved grip strength, pinch strength, thumb abduction degree (palmar and radial), and Kapandji opposition scores. The subjective evaluation involved visual analog scale (VAS) and Disabilities of the Arm, Shoulder, and Hand (DASH) scores.

**Results:**

Intergroup differences in pinch strength, thumb abduction degrees (palmar and radial), and Kapandji opposition scores were obvious, whereas those in grip strength, VAS score, and DASH score were not.

**Conclusion:**

In Chinese patients, both techniques relieved pain and improve grip strength. Arthrodesis displayed better pinch strength, while arthroplasty displayed better motor function. Patients were satisfied with the effects of both techniques.

## Background

The thumb carpometacarpal (CMC) joint is a saddle joint with both concave and convex surfaces and a thin and loose capsule. Due to the effect of constant multidirectional forces during daily work and life activities, about 25% of females and 12% of males suffer from thumb CMC osteoarthritis in the west [1]. Symptoms of thumb CMC osteoarthritis include pain, swelling, deformity, and instability. Multiple methods are used to treat progressive thumb CMC osteoarthritis, among which trapeziometacarpal arthrodesis and trapezial excision with ligament reconstruction and tendon interposition (LRTI) are the most common. These two surgical treatment methods have received mixed reviews in previous studies in the west patients. This retrospective study studied the results of 39 Chinese patients of progressive thumb CMC osteoarthritis treated with either arthrodesis or arthroplasty. Through the evaluation of grip strength, pinch strength, thumb abduction angles (palmar and radial), Kapandji opposition scores, visual analog scale (VAS) scores, and Disabilities of the Arm, Shoulder, and Hand (DASH) scores, the two groups of data were compared to observe the degree of improvements of the two surgical treatments in strength, pain, range of motion, and overall satisfaction. The following is a report of this study.

## Methods

### General materials

Between February 2012 and September 2017, a total of 43 Chinese patients with stage II or III thumb carpometacarpal osteoarthritis underwent surgery (trapeziometacarpal arthrodesis in 24, trapezial excision with LRTI in 19). In the group, 4 patients were lost for follow-up (arthrodesis 2 and LRTI 2), these 4 patients were not enrolled in the data. There were 39 Chinese patients in this study included 22 of arthrodesis and 17 of arthroplasty (12 males, 27 females; 11 cases of left hand, 28 cases of right hand, 25 cases of dominant hand, and 14 cases of non-dominant hand). The age distribution was as follows: 40–49 years, 4 cases; 50–59 years, 12 cases; 60–69 years, 18 cases; and 70–79 years, 5 cases. All diagnoses were degenerative thumb CMC osteoarthritis. With the exception of traumatic arthritis, the X-ray films of patients prior to surgery were sorted by Eaton-Glickel classification [2] into 12 cases of stage II and 27 cases of stage III.

The surgical procedure performed was determined by a shared decision between the patient and the surgeon based on a standardized description of advantages and disadvantages of each procedure. Surgeons were not restricted from additional presurgical counseling, and each patient ultimately elected the operative treatment of his or her choice.

### Surgical methods

#### Arthrodesis

**Fig. 1:**
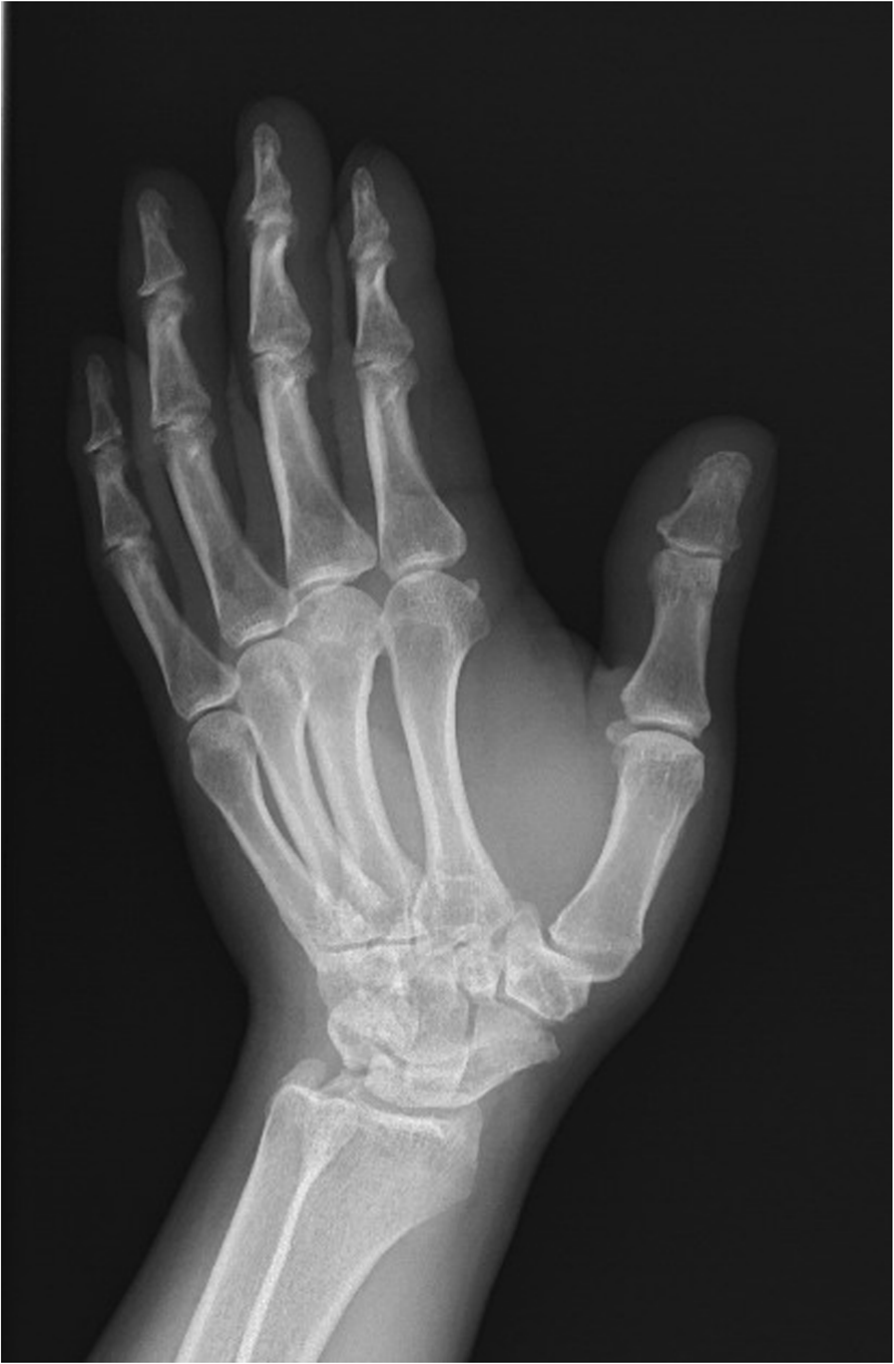
Arthrodesis preoperation

**Fig. 2:**
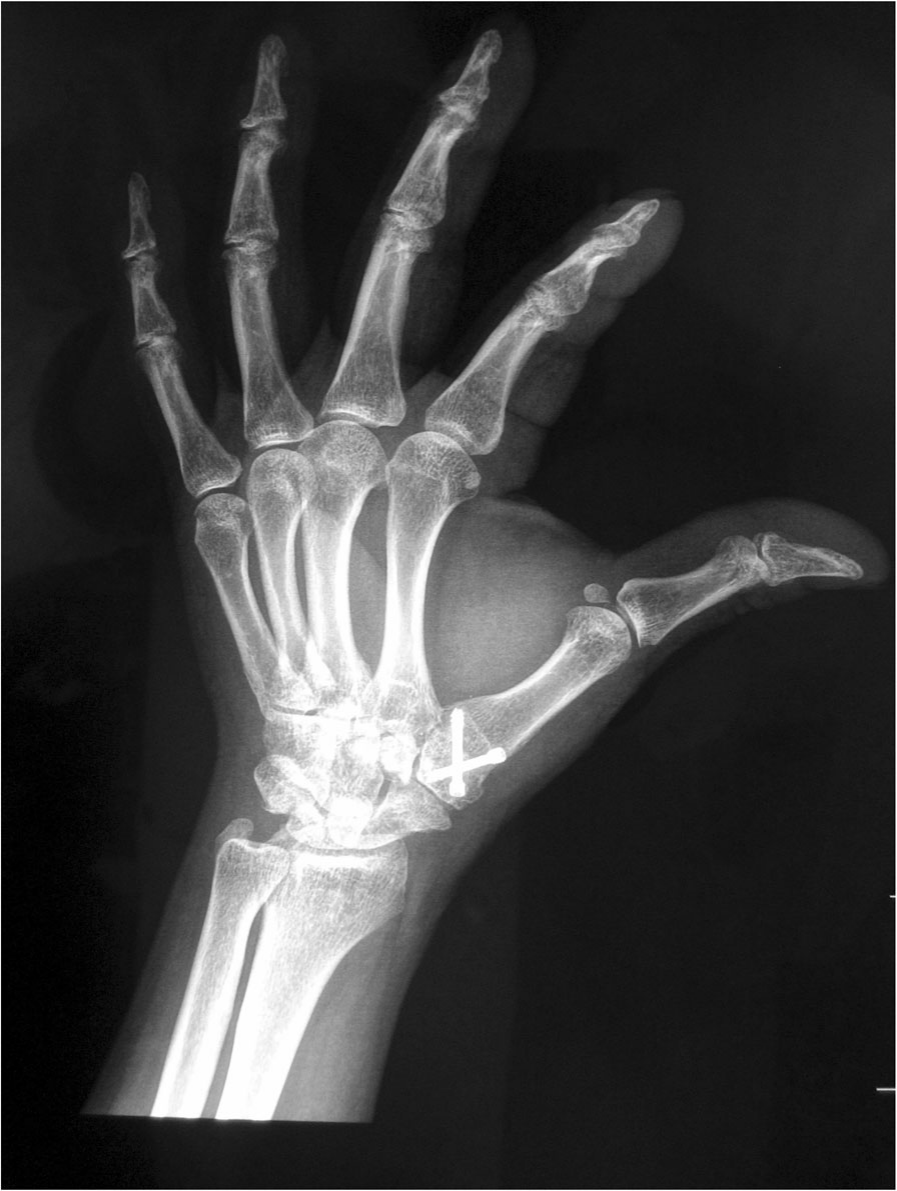
Arthrodesis postoperation

An S-shaped incision was made on the radiodorsal side of the thumb CMC joint and the cutaneous nerves were protected. The extensor pollicis brevis and abductor pollicis longus muscle tendons were pulled toward either side. The dorsal joint capsule of the thumb CMC joint was cut open, osteophytes and synovial membrane were removed, and articular cartilage was removed to expose cancellous bone. Cannulated screws were used to fuse the trapezium and the base of the first metacarpal. The thumb position of fusion was at 35° of radial abduction, 35° of palmar abduction, 15° of pronation, and 10° of dorsiflexion [3]. After the procedure, the joint capsule, subcutaneous tissues, and skin were closed layer by layer. To avoid over-shortening of the thumb when adjusting angle and to increase the rate of successful joint fusion in patients with lower bone quality, 8 patients (age ≥ 65) underwent bone transplantation using bone grafts harvested from the styloid process of the radius. After 2 weeks of postoperative external plaster fixation, rehabilitation training was conducted under the guidance of a physical therapist (Figs. 1 and 2).

#### Arthroplasty

An incision was made on the radiodorsal side of thumb CMC joint and the cutaneous nerves and extensor muscle tendons were protected. The thumb CMC joint capsule was cut open and the trapezium was removed. A bone tunnel was drilled at the base of the first metacarpal; 8 cm of the flexor carpi radialis tendon was transected by half of its width, flipped toward the distal end, passed through the bone tunnel, tightened and fixated at the base of the first metacarpal, and then sutured back onto itself. The rest of the tendon was rolled and sutured into a “tendon ball” shape and used to fill the wrist defect caused by the trapeziectom y[4], while a K-wire was used to hold the first metacarpal in the abduction position. The joint capsule, subcutaneous tissues, and skin were then closed layer by layer and external plaster fixation was implemented. At 6 weeks postoperative, the K-wire and plaster were removed, and the functional training was conducted under the guidance of a physical therapist.

### Index of observation

Follow-up visits were conducted at 2 and 4 weeks as well as at 3, 6, and 12 months and then every 6 months thereafter. Doctor visits could be made any time when discomfort was experienced without time constraints. The follow-up clinical indices included grip strength, pinch strength, abduction angles (palmar and radial), Kapandji opposition score, VAS pain score, and DASH score. Grip and pinch strength are largely affected by sex, age, occupation, and handedness; therefore, they were analyzed using the percentage of postoperative strength growth to increase objective accuracy.

## Measurement methods

Grip strength, pinch strength, abduction angles (palmar and radial), and Kapandji score were measured and recorded using a Biometrics E-LINK Hand Kit (H500 evaluation system, X4 data adapter). Distal-lateral phalangeal pinching was used in the pinch strength measurement. Each patient was measured 3 times and the average score was recorded. The VAS pain score and DASH score were evaluated using questionnaires.

### Statistical analysis

Clinical follow-up visit indices were statistically processed and analyzed using SPSS 16.0 statistical software. The data are shown as mean ± standard deviation. Measurement data were analyzed using the *t* test according to data characteristics, with values of *P* < 0.05 considered statistically significant.

## Results

### General results

Follow-up visits were conducted for all 39 patients for a mean 2.5 years (range, 12 months to 4 years). At the 12 months follow-up, all patients were satisfied with their treatment outcomes. Thumb base pain disappeared and grip and pinch strengths increased. The complications discovered during follow-up were primarily cutaneous nerve damage, with 5 cases of numbness at the surgical site. No infections of the incision or deep tissues occurred. All arthrodesis cases achieved osseous fusion 3 months postoperative and no cases of nonunion occurred.

### Postoperative functional evaluation

Preoperative and 12-month postoperative grip strength, pinch strength, abduction angles (palmar and radial), Kapandji opposition scores, and VAS and DASH scores are shown in Tables 1, 2 and 3.

**Table 1:** Pre- and 12-month postoperative grip and pinch strength test results

	*n*	Grip strength			Lateral pinch strength		
		Preoperative (kg)	Postoperative (kg)	Increase (%)	Preoperative (kg)	Postoperative (kg)	Increase (%)
Arthrodesis	22	15.2 ± 7.25	25.2 ± 6.21	65.5 ± 3.2	5.2 ± 1.6	7.8 ± 1.8	51.1 ± 5.5
Arthroplasty	17	14.8 ± 7.03	24.6 ± 7.31	63.2 ± 4.1	5.6 ± 1.9	7.2 ± 1.7	39.8 ± 6.2
*t*				1.692			5.389
*P*				0.18			< 0.001

Values are shown as mean ± standard deviation

**Table 2:** Pre-surgery and 12-month post-surgery palmar and radial abduction angles (mean ± standard deviation)

	*n*	Palmar abduction		Radial abduction	
		Preoperative (°)	Postoperative (°)	Preoperative (°)	Postoperative (°)
Arthrodesis	22	52.6 ± 8.3	41.6 ± 5.7	45.2 ± 5.2	39.2 ± 4.3
Arthroplasty	17	54.8 ± 7.2	58.9 ± 7.3	46.6 ± 7.2	52.6 ± 3.6
*t*			6.021		5.917
*P*			< 0.001		< 0.001

Values are shown as mean ± standard deviation

**Table 3:** Pre- and 12-month postoperative VAS, Kapandji, and DASH scores

	*n*	VAS		Kapandji score		DASH score	
		Preoperative	Postoperative	Preoperative	Postoperative	Preoperative	Postoperative
Arthrodesis	22	5.5 ± 1.7	0.4 ± 0.1	7.2 ± 0.6	6.7 ± 0.8	41.2 ± 6.2	4.2 ± 1.2
Arthroplasty	17	5.2 ± 1.3	0.4 ± 0.2	6.9 ± 0.5	7.5 ± 1.2	39.8 ± 6.7	4.3 ± 1.8
*t*			1.431		3.351		1.745
*P*			0.25		0.004		0.23

*VAS*, visual analog scale; *DASH*, Disabilities of the Arm, Shoulder, and Hand

Values are shown as mean ± standard deviation

#### Grip strength

The arthrodesis and arthroplasty groups demonstrated significant increases in grip strength 12 months postoperative with increased percentages of 65.5 ± 3.2% and 63.2 ± 4.1%, respectively. However, the difference in grip strength increase between the two groups was *P* = 0.18, indicating no statistical significance despite improved grip strength in both groups.

#### Pinch strength

The arthrodesis and arthroplasty groups showed significant increases in pinch strength 12 months postoperative of 51.1 ± 5.5% and 39.8 ± 6.2%, respectively. The difference in pinch strength increase was *P* < 0.001, indicating that arthrodesis was more effective than arthroplasty at improving pinch strength.

#### Palmar abduction

Compared with preoperative, the palmar abduction of arthrodesis patients decreased from 52.6 ± 8.3° to 41.6 ± 5.7° at 12 months postoperative with a mean decrease of about 10°. Compared with preoperative, the palmar abduction of arthroplasty patients increased from 41.6 ± 5.7° to 58.9 ± 7.3° with a mean increase of about 5°. The difference in palmar abduction decrease was *P* < 0.001, indicating that arthroplasty can better improve the range of motion of thumb palmar abduction than arthrodesis.

#### Radial abduction

Compared with preoperative, the radial abduction of arthrodesis patients decreased from 45.2 ± 5.2° to 39.2 ± 4.3° at 12 months postoperative with a mean decrease of about 6°. Compared with preoperative, the radial abduction of arthroplasty patients increased from 46.6 ± 7.2° to 52.6 ± 3.6° at 12 months postoperative with a mean increase of about 6°. The comparison of the two surgical methods resulted in a *P* < 0.001, indicating that arthroplasty can better improve the range of motion of thumb radial abduction.

#### VAS pain score

In the arthrodesis group, the score decreased from 5.5 ± 1.7 preoperative to 0.4 ± 0.1 postoperative; the pain mainly disappeared or only a slight pain was experienced. In the arthroplasty group, the score decreased from 5.2 ± 1.3 preoperative to 0.4 ± 0.2 postoperative; the pain also mainly disappeared or only a slight pain was experienced. The comparison between the two groups resulted in a value of *P* = 0.25, indicating no statistically significant difference in pain relief between the two surgical methods.

#### Kapandji opposition scores

In the arthrodesis group, the mean score decreased from 7.2 ± 0.6 preoperative to 6.7 ± 0.8 postoperative, suggesting a slight decline in thumb opposition function. In arthroplasty group, the mean score increased from 6.9 ± 0.5 preoperative to 7.5 ± 1.2 postoperative, suggesting a slight increase in thumb opposition function. The intergroup comparison resulted in a value of *P* = 0.004, indicating that arthroplasty can better improve thumb opposition function than arthrodesis.

#### DASH score

In the arthrodesis group, the mean score decreased from 41.2 ± 6.2 preoperative to 4.2 ± 1.2 postoperative, suggesting a very slight impact on postoperative upper-limb function. In the arthroplasty group, the mean score decreased from 39.8 ± 6.7 preoperative to 4.3 ± 1.8 postoperative, also suggesting a very slight impact on postoperative upper-limb function. The intergroup comparison of final DASH scores revealed a value of *P* = 0.23, indicating that both surgical methods can largely improve upper-limb function and no statistically significant difference was found in patient satisfaction.

## Discussion

The selection of treatment plans for thumb CMC osteoarthritis is primarily based on illness stage [5]. For early-stage CMC osteoarthritis, conservative treatments such as adjusting daily activities, splint fixation, and local corticosteroid injections can be utilized to relieve symptoms. Wrist arthroscopy can be used to clear intra-articular osteophytes and thickened synovial membranes, perform hemitrapeziectomy [6, 7] and soft tissue interposition [8], and conduct joint capsule thermal shrinkage therapy [9]. However, its long-term therapeutic outcomes are inconclusive. An elastic silicone prosthetic implant is currently rarely used in thumb CMC joint replacement due to reactive synovitis and prosthetic wear [10]. The use of Artelon implant is not recommended because of its high revision rate and worse outcomes compared with conventional techniques [11]. Similarly, spherical polymer implant failures have been reported [12]. Recently, a report of a pyrocarbon spherical (Pi2) implant demonstrated high complication rates and no identifiable benefit [13]. For progressive-stage (stage II or III) thumb CMC osteoarthritis, arthrodesis and arthroplasty are currently widely used and their effect is positive. Therefore, a comparison and evaluation of the effects of the two methods are necessary.

In this study, arthrodesis and arthroplasty were able to significantly improve patient grip and pinch strengths. Comparing the two methods resulted in no statistically significant difference in grip strength improvement (*P* = 0.18, *P* > 0.05), while lateral pinch strength showed better improvement after arthrodesis (*P* < 0.001). The force couple in gripping motion has a shorter moment arm, while the force couple in pinching motion has a longer moment arm that causes higher joint compression strength [14]. This result indirectly proved that arthrodesis tends to better improve joint stability than arthroplasty.

Arthrodesis can significantly relieve pain in cases of degenerative thumb CMC osteoarthritis [15]. The literature suggests its main disadvantage being decreased postoperative thumb range of motion, with the thumb unable to adduct and lay flat on the palm, as well as a certain rate of nonunion [16, 17]. We observed a decrease in thumb abduction angle postoperative; however, the joint was not completely fixed and was unable to move. Kapandji scores reached 6.7 postoperative, which was only a slight decrease from 7.2 prior to surgery. Other studies also indicated that simple CMC arthrodesis will not affect the motor functions of the thumb since the range of motion in the trapezium–first metacarpal joint postoperative is compensated for by the ranges of motion of the scaphoid–trapezium–trapezoid joint and first metacarpal–proximal phalanx joint. The majority (75%) of the compensation comes from the first metacarpal–proximal phalanx joint, while the other 25% comes from the scaphoid–trapezium–trapezoid joint [18]. In follow-up visits, we observed thumb adduction being affected due to CMC arthrodesis fixating the joint. However, it was rarely noticed by patients and did not affect their overall satisfaction.

After thumb trapeziometacarpal arthrodesis surgery, the surrounding joints will compensate for its movement, thereby increasing the rates of osteoarthritis in these joints [19]. In 22 arthrodesis patient follow-up visits, we discovered 2 cases of scaphoid–trapezium–trapezoid osteoarthritis and 1 case of thumb metacarpal–proximal phalanx osteoarthritis. However, they all presented only on X-ray radiographs without subjective symptoms; therefore, no further treatment was given [20].

Nonunion after trapeziometacarpal arthrodesis was once considered the major complication of arthrodesis. The literature has reported a 13–16% nonunion rate [17]. We utilized Cannulated screws in all 22 patients to fix the joint; stability and compression effectiveness of joint fusion were significantly increased compared with K-wires [21]. In some patients with lower bone quality, we used bone grafts harvested from the styloid process of the radius, further increasing the arthrodesis fusion rates. All patients in this group achieved 100% union.

**Fig. 3:**
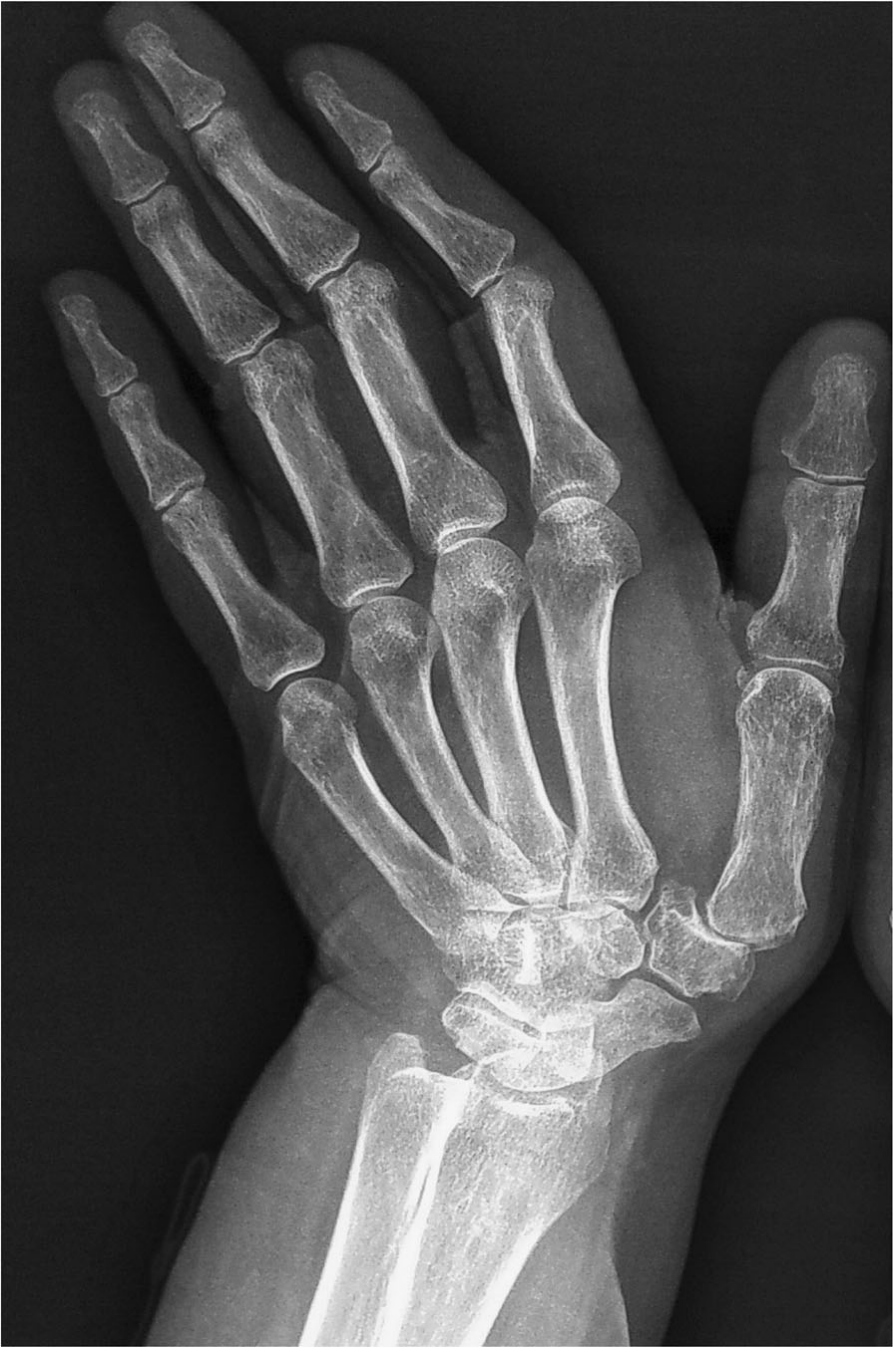
LRTI preoperation

**Fig. 4:**
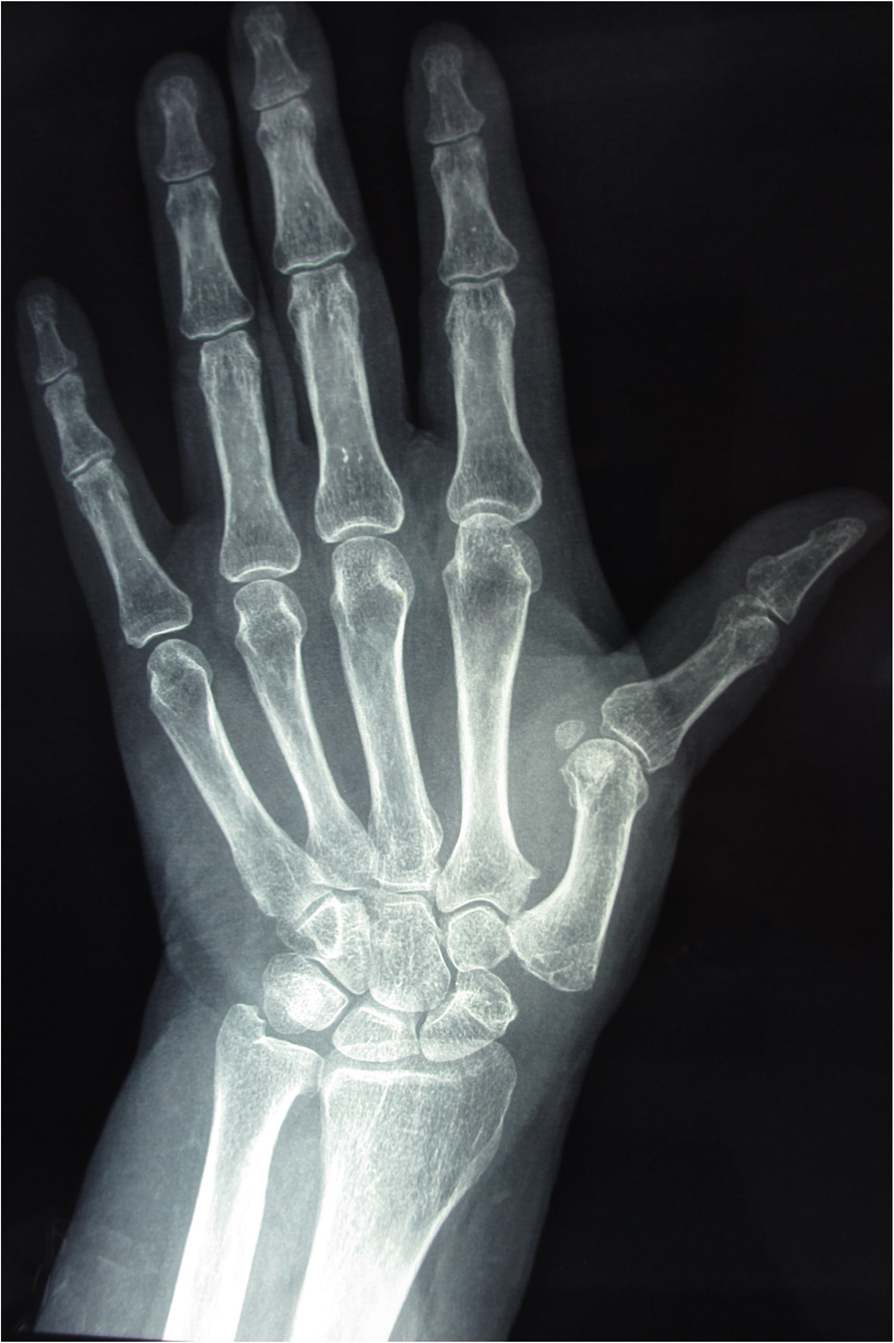
LRTI postoperation

LRTI can significantly relieve pain because it involves removal of the affected joint. In this study, the thumb abduction angle increased after arthroplasty surgery. Palmar abduction increased by 4°, radial abduction increased by 6°, and Kapandji score increased from 6.9 preoperative to 7.5 postoperative. Patients noted relatively flexible thumbs. However, although ligament reconstruction and suspension were utilized and tendon interposition was used to support the first metacarpal, postoperative thumb shortening and instability were present [22], affecting pinch strength (compared with pinch strength after arthrodesis, *P* < 0.01). Kapandji score did not reach the normal level of 10 (Figs. 3 and 4).

Pain is the main reason why patients decide to undergo surgical treatment. The pain VAS is an important postoperative evaluation index. Both groups of patients experienced moderate pain with a VAS score of 5. Their pain affected sleep and pain relief medication was necessary. After arthrodesis and arthroplasty surgery, patient pain disappeared or there was only slight pain experienced during movement (VAS = 0.4). The intergroup comparison resulted in a value of *P* = 0.25, indicating no statistically significant difference in pain relief between the two surgical methods.

Thumb grip strength, abduction angle (palmar and radial), and Kapandji scores all evaluate hand functions from iatrogenic perspectives and thus cannot fully reflect patients’ subjective feelings. DASH scores evaluate upper-limb function from the patients’ subjective perspective; thus, they are more persuasive. In this study, DASH scores in both groups decreased from 41.2 ± 6.2 and 38.8 ± 6.7 preoperative to around 4 postoperative, respectively. No statistically significant difference was found in the comparison of final scores between the two groups (*P* = 0.23, *P* > 0.05), indicating that the patients were able to ignore the differences in joint mobility and thumb strength and were satisfied with the therapeutic outcomes of both surgical methods. This suggests that upper-limb function after arthrodesis and arthroplasty surgeries have no significant impact on daily life.

In this study, all the case data were from Chinese. Previous literature reported that Trapezial-metacarpal (TM) joint surfaces appear to be shallower in Asian than in white race, and the frequency of TM osteoarthritis seems to be substantially lower in Asian TM joints [23]. Due to different anatomical factors, the results of surgery may be different. The study represents the results of surgery in the Chinese population. This is a supplement to previous data.

## Conclusions

In Chinese patients, although differences existed between the therapeutic outcomes of arthrodesis and arthroplasty on some objective evaluation indices, our results showed subjective patient satisfaction with both surgical treatments. Therefore, personalized treatment plans can be designed according to patients’ demands for strength and flexibility.

## Data Availability

Not applicable.
